# Tumor Cell Targeting by Iron Oxide Nanoparticles Is Dominated by Different Factors *In Vitro* versus *In Vivo*

**DOI:** 10.1371/journal.pone.0115636

**Published:** 2015-02-19

**Authors:** Christian NDong, Jennifer A. Tate, Warren C. Kett, Jaya Batra, Eugene Demidenko, Lionel D. Lewis, P. Jack Hoopes, Tillman U. Gerngross, Karl E. Griswold

**Affiliations:** 1 Thayer School of Engineering, Dartmouth, Hanover, NH, United States of America; 2 Department of Biostatistics and Medicine, The Geisel School of Medicine at Dartmouth, Lebanon, NH, United States of America; 3 Program in Molecular and Cellular Biology, Dartmouth, Hanover, NH, United States of America; 4 Department of Biological Sciences, Dartmouth, Hanover, NH, United States of America; 5 Department of Chemistry, Dartmouth, Hanover, NH, United States of America

## Abstract

Realizing the full potential of iron oxide nanoparticles (IONP) for cancer diagnosis and therapy requires selective tumor cell accumulation. Here, we report a systematic analysis of two key determinants for IONP homing to human breast cancers: (i) particle size and (ii) active vs passive targeting. *In vitro*, molecular targeting to the HER2 receptor was the dominant factor driving cancer cell association. In contrast, size was found to be the key determinant of tumor accumulation *in vivo*, where molecular targeting increased tumor tissue concentrations for 30 nm but not 100 nm IONP. Similar to the *in vitro* results, PEGylation did not influence *in vivo* IONP biodistribution. Thus, the results reported here indicate that the *in vitro* advantages of molecular targeting may not consistently extend to pre-clinical *in vivo* settings. These observations may have important implications for the design and clinical translation of advanced, multifunctional, IONP platforms.

## Introduction

Advances in nanotechnology are now driving a revolution in cancer detection and treatment, and iron oxide nanoparticles (IONPs) were some of the first nanomaterials to see application in oncology. In the field of bioimaging, IONPs have seen extensive application as contrast agents for magnetic resonance imaging (MRI) [1–6]. With respect to therapy, studies by Gilchrist *et al*. in the mid-20^th^ century suggested that lymphatic metastasis could be therapeutically heated by activating localized IONPs *via* an alternating magnetic field [7]. In the decades following this initial proof of concept study, other groups have successfully implemented this treatment modality both *in vitro* [8–16] and *in vivo* [8–12]. In addition to killing cancer cells directly, hyperthermia can enhance the efficacy of radiation and chemotherapies[17,18] and can indirectly stimulate the innate anti-cancer immune response [19,20]. Ultimately, however, the therapeutic index (defined in humans as the TD_50_/ED_50_) of IONP therapies and the imaging sensitivity of IONP contrast agents is a function of differential particle concentrations at sites of malignancy versus healthy tissues.

In the case of nanoparticles that lack targeting moieties, tumor accumulation is dependent upon direct injection, selective tumor embolization, or passive targeting as a result of uptake by either the reticuloendothelial system or the enhanced permeability and retention (EPR) effect [4,17,18,21]. With more advanced platforms, nanoparticles may be actively targeted to cancer cells by surface functionalization with various moieties. Examples include natural ligands for cell surface receptors, small molecules, nucleic acids, carbohydrates, peptides, and non-immunoglobulin scaffolds [4,21]. To date, however, antibodies have been the most widely used targeting ligands [22–25]. Monoclonal antibodies (mAb) have shown particular promise in localizing IONP for *in vivo* magnetic hyperthermia [26,27]. Of even greater relevance to the current study, Trastuzumab (Tmab; Herceptin) has been used to target IONP to human breast cancers *in vitro* and *in vivo*, although the latter success required direct tumor injection[28–32]. However, further studies are needed to fully understand key parameters that control the efficiency with which IONP selectively home to cancer cells following systemic administration *in vivo*.

While yielding promising preliminary results, mAbs have inherent limitations as nanoparticle targeting agents. With an average molecular weight of 160,000 daltons and diameters of up to 15 nm, full length IgG antibodies are relatively large molecules that can significantly alter IONP size and surface properties, thereby diminishing selective tumor localization and diagnostic/therapeutic utility[4]. Additionally, the Fc portion of full length antibodies has the potential to compromise tumor specificity due to off-target binding by various Fc receptors expressed in healthy tissues [33,34]. Smaller, engineered antibody fragments directly address some of these inherent limitations. To better capitalize on active IONP targeting for human breast cancer, the studies described here employed a monovalent Fab’ fragment of Tmab (referred to hereafter as Tfab). A systematic approach was applied to nanoparticle design, functionalization, *in vitro* characterization, and analysis of *in vivo* biodistribution. This controlled, comparative study yields new insights into the relationships between IONP size, molecular targeting, surface functionalization, and accumulation on human breast cancer cells.

## Methods

For cell line information, see S1 File.

### Tfab conjugation to 30 nm and 100 nm iron Oxide Nanoparticles (IONPs)

Trastuzufab (Tfab) protein sequence was reformatted from its corresponding and commercial full IgG molecule, Trastuzumab (trade name, Herceptin) (Tmab) protein sequence available from literature. CMVR VRC01 expression vectors (NIH AIDS reagent program, Germantown, MD) separately harboring Tfab light chain and heavy chain were co-transfected into suspension HEK 293 cells and purified using Kappa select and superdex 75 chromatography columns (GE Healthcare, Pittsburgh, PA). Reductive activation and chemical conjugation of purified Tfab were performed as described in supplemental methods (S1 File).

30 nm and 100 nm aminodextran coated IONPs were purchased from BioPal (Worcester, MA) and Micromo Partikeltechnologie GmbH (Germany), respectively. To perform site conjugation, Sulfo GMBS (Thermo Scientific, Rockford, IL) was added to IONPs and incubated at room temperature for 2 hours. Cysteine reduced Tfab was added to the activated IONP at an equal w/w ratio and incubated at room temperature for 16 hours at 4°C. All process was performed in a sterile environment using sterile and endotoxin free buffers. For PEGylation, PEG thiol (Laysan Bio, AL) average molecular weight was reduced with TCEP and assayed by the barium chloride/iodine method[35]. Mixed PEGylated Tfab and IONPs were prepared as described for non-PEGylated IONPs (see supporting information for details).

### 30 nm and 100 nm Tfab functionalized Nanoparticles binding studies

Quantification of the number of Tfab/IONPs was performed as described in supplemental methods (S1 File). The rHER2-his (AcroBiosystems, Bethesda, MD) and cells (SKBR3 and BT-474) binding studies procedures of 30 nm and 100nm Tfab functionalized nanoparticles are described in details in supplemental methods (S1 File).

### BT-474 tumor model

All mice were cared for according to approved Dartmouth College Institutional Animal Care and Use Committee (IACUC) animal protocol (protocol number hoop.pj.8). This study was approved by the Dartmouth College IACUC. All efforts were made to minimize animals suffering. NOD.Cg-Prkdcscid Il2rgtm1Wjl/SzJ (NSG) mice were obtained from Jackson Laboratories or an in-house stock originally from Jackson Laboratories. At 8–11 weeks old, mice were implanted below the mammary fat pad with 5 million BT-474 cells in 100 μl of a three part mixture of rat tail collagen I (BD Biosciences, San Jose, CA), Matrigel^TM^ basement membrane matrix (BD Biosciences, San Jose, CA) and serum-free DMEM-F12 50/50 using a 1ml syringe and a 30G needle. Mice were put on study upon reaching a tumor volume of 100–200mm^3^, as measured by calipers and calculated using an ellipsoid approximation.

### 30 nm and 100 nm Tfab functionalized Nanoparticles administration

IONP dose was calculated using mouse body mass (g). Doses of 0.08 mg Fe/g mouse were used and IONPs-Tfab stocks were diluted with PBS to the target concentration immediately prior to injection. A 1ml syringe with 30g needle was primed and loaded with the full injection dose, and half the volume marked off. Mice were anesthetized using isoflurane and secured on a heated, vented surface with an isoflurane nose cone for injection. Half the IONP dose or an equivalent volume of PBS was injected intravenously into the tail vein; the mouse was then allowed to wake up on the heated surface, and was then returned to her cage. Two hours following the first injection, the syringe was drawn back to recover any void volume in the needle and the needle was replaced with a fresh tip. The mouse was then re-anesthetized and injected with the second half of the IONP dose. 24 hours after IONPs injection, mice were euthanized according to approved protocol with an overdose of isoflurane and checked for pain stimulus response. Tissues harvesting and ICP-MS digestion were performed as described in supplemental methods (S1 File).

## Results

### Design, Production, and Analysis of Tfab

**Fig 1:**
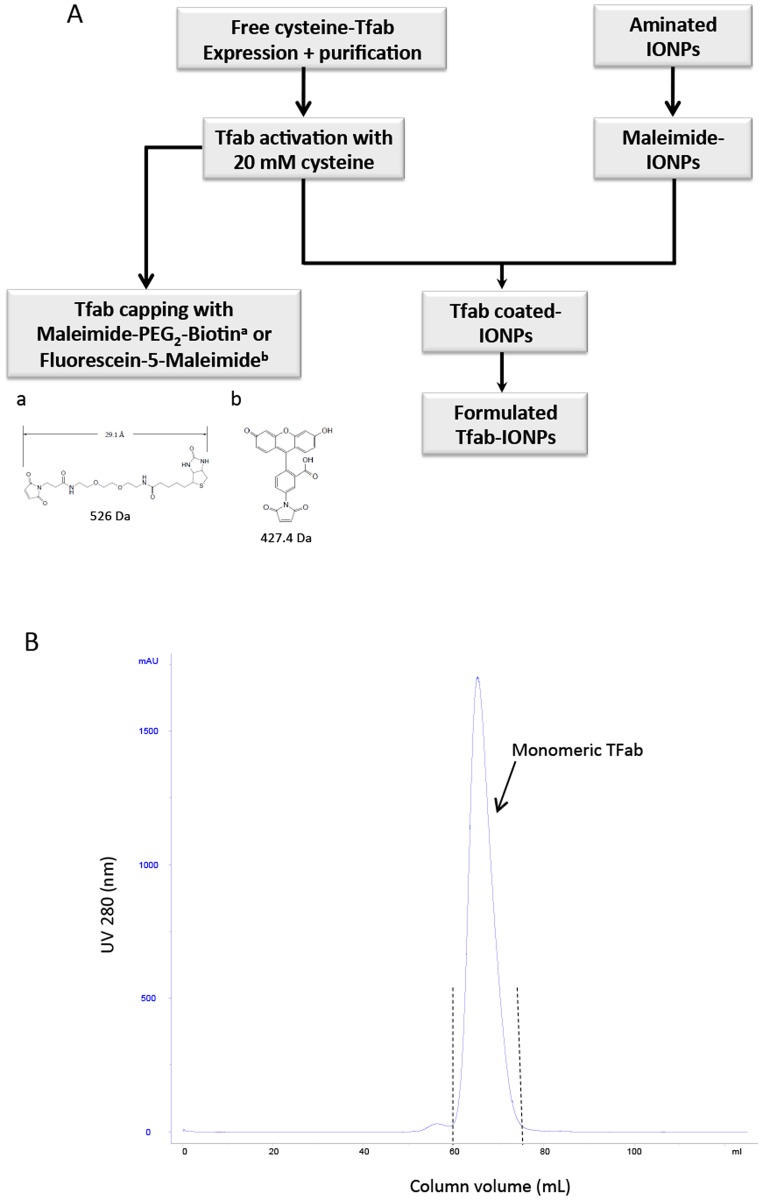
Purification and characterization of Tfab antibody fragment. (A) Schematic illustration of workflow for Tfab and IONP functionalization. Monomeric Tfab is subjected to reduction/activation using 20 mM cysteine followed by conjugation with Maleimiede-PEG2-Biotin, Fluorescein-5-Maleimide, or Maleimide-IONP. (B) Size exclusion purification chromatogram. The blue curve represents UV absorbance at 280 nm and dashes lines represent the collected monomeric Tfab fraction.

To minimize the molecular targeting moiety’s contribution to overall IONP size, an antigen binding fragment (Fab’) of the FDA approved monoclonal antibody Tmab was engineered as described in the methods section. The workflow for functionalization and conjugations of the engineered antibody and IONPs is shown in Fig. 1A. This antibody fragment, Tfab, was constructed such that the heavy chain’s C-terminal cysteine (residue 229) was left unpaired (S1B Fig.). This design ultimately enabled site specific conjugation of the Tfab to maleimide-PEG_2_-Biotin, fluorescein-5-maleimide or maleimide-IONPs. The desired monomeric Tfab fraction was isolated by Kappa Select affinity purification and size exclusion chromatography of recombinant Tfab expressed in HEK 293 cells (Fig. 1B).

### Tfab specifically binds to recombinant HER2 and HER2 expressing tumor cells

To enable functional analysis of the purified Tfab, the free cysteine of the heavy chain was capped with a hetero bifunctional maleimide-PEG_2_-biotin of 526 Da. Upon reaction with the biotin moiety, mass spectral analysis showed the Tfab base peak shifted from 47984 Da to 48510 Da, consistent with the addition of a single maleimide-PEG_2_-biotin molecule (S1F Fig.). Binding of maleimide-PEG_2_-biotin labeled Tfab to recombinant HER2 (rHER2) was initially analyzed by ELISA. As a performance benchmark, analogous studies were conducted with commercially sourced Tmab IgG (Herceptin). The engineered Tfab fragment and the full length IgG antibody (Tmab) exhibited equivalent sub-nanomolar EC_50_ values (Table 1 and S2A Fig.). Additionally, serial dilutions of the Tfab-Maleimide-PEG_2_-biotin were employed in rHER2 competition binding experiments against a single saturating concentration of Tmab IgG (Table 1 and S2B Fig.). The engineered Tfab displaced Tmab binding with low nanomolar IC_50_, indicating that it retained the same epitope specificity as the full length parental IgG. Subsequently, more detailed rHER2 binding kinetics were analyzed using biolayer interferometry (Table 1 and S3 Fig.). Tfab-maleimide-PEG_2_-biotin and Tmab were immobilized on streptavidin and rprotein A biosensors tips, respectively, and then assayed with rHER2-his protein. While the on rates of both antibodies were similar, Tfab exhibited a 10-fold slower apparent off rate compared to the parental IgG. As a result, the engineered Tfab possessed a marginally improved apparent equilibrium dissociation constant relative to full length Tmab (K_D_ = 0.19 nM versus 1.0 nM, respectively). It is likely that this small difference resulted from the high affinity biotin-streptavidin capture interaction for Tfab versus the lower Protein A-Fc capture interaction for Tmab IgG. Importantly, no binding of Tfab-maleiemide-PEG_2_-biotin or Tmab was observed when rHER3 protein was used as analyte in either ELISA or bio-layer interferometry experiments (S2 and S3 Figs.). In aggregate, the quantitative binding studies with rHER2 demonstrated that the engineered Tfab has the same epitope specificity and monovalent binding affinity as its full length IgG counterpart.

**Table 1:** *In vitro* binding affinity of Tfab and Tmab.

	Tfab	Tmab
EC_50_ rHER2 (nM)	0.1	0.1
IC_50_ rHER2* (nM)	12	N/A
k_on_ (M^-1^s^-1^)	1.42E+05 ± 500	3.03E+05 ± 1000
k_off_ (s^-1^)	2.64E-05 ± 2E-06	3.03E-04 ± 2. E-06
K_D_ (nM)	0.19 ± 0.01	1.00 ± 0.01

*IC_50_ values reflect concentration of Tfab required to compete 50% of saturating Tmab IgG.

Errors are standard deviations from technical triplicates.

While the above *in vitro* studies demonstrated that Tfab-maleimide-PEG_2_-biotin efficiently bound both immobilized and soluble rHER2, the ultimate *in vivo* utility of targeted IONP is dependent upon antibody recognition of the HER2 receptor in its biological context. To assess cellular binding, the free heavy chain cysteine of purified and activated Tfab was capped with fluorescein-5-Maleimide, and the fluorescent conjugate (Tfab-fluorescein-5-Maleimide) was assayed by flow cytometry with a panel of live human cancer cells exhibiting either high or low HER2 expression levels (S4 Fig.). After one hour incubation, Tfab-fluorescein-5-Maleimide was found to exhibit low nanomolar binding affinity for both BT-474 and SKBR3 breast cancer lines (Table 2 and S2C Fig.), each of which expresses high levels of HER2. In contrast, no Tfab-fluorescein-5-Maleimide binding was detected on live MCF7 (S2C Fig.), a breast cancer cell line that expresses lower levels of HER2. Likewise, no binding was observed with live ovarian cancer cell lines SKOV3 or A2780, both of which were found to express negligible levels of HER2. Furthermore, serial dilutions of Tfab-maleimide-PEG2-biotin were employed in cellular competition binding studies (using BT-474 and SKBR3) against a single saturating concentration of Tmab. Similar to the *in vitro* competition ELISA studies, the observed low nanomolar IC_50_ values demonstrated that the engineered antibody fragment bound the same cellular epitope as the full length IgG (Table 2 and S2D Fig.). As a whole, these data demonstrated that Tfab efficiently and specifically targeted cancer cells in a HER2-dependent fashion.

**Table 2:** Tfab binding performance with breast cancer cell lines.

Cell line	EC_50_ (nM)	IC_50_ (nM)*
SKBR3	4	12
BT-474	3	13

*IC_50_ values reflect concentration of Tfab required to compete off 50% of a saturating Tmab IgG.

### Construction and analysis of Tfab functionalized IONP

**Fig 2:**
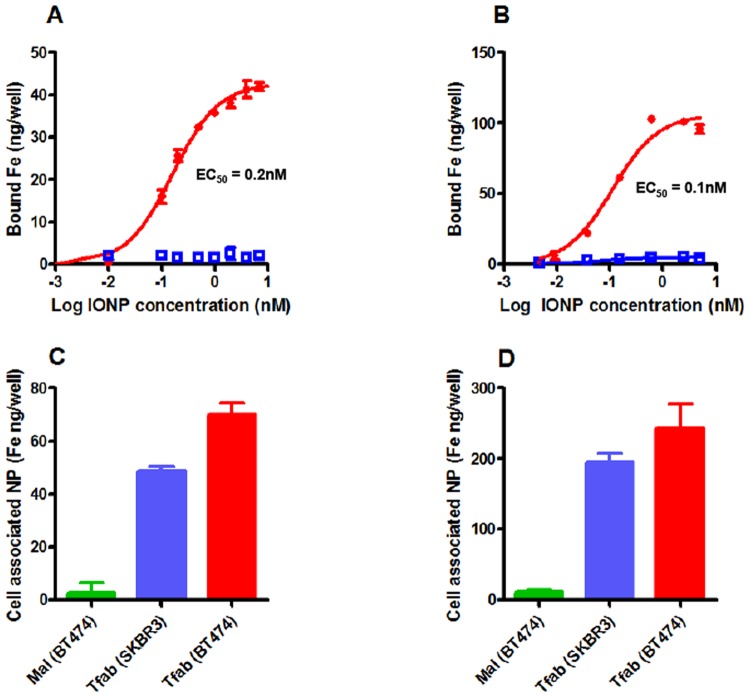
*In vitro* binding studies of IONP. (**A**) Dose response rHER2 binding curves for 30 nm IONP-Tfab (closed circles) and 30 nm IONP-Mal (open squares). (**B**) Dose response rHER2 binding curves for 100 nm IONP-Tfab (closed circles) and 100 nm IONP-Mal (open squares). (**C**) Binding of HER2+ breast cancer cells for 30 nm IONP-Tfab and 30 nm IONP-Mal, both dosed at 100 μg/ml. (**D**) Binding of HER2+ breast cancer cells for 100 nm IONP-Tfab and 100 nm IONP-Mal, both dosed at70 μg/ml. Error bars represent standard deviation.

Having validated Tfab specificity and high affinity for the HER2 receptor, the antibody fragment was conjugated to both small and large maleimide-IONP. These conjugations yielded matched IONP-Tfab constructs that differed only in their approximate size (~30 nm versus ~100 nm diameter, respectively) and number of Tfab targeting moieties (~10 versus ~90, respectively; Table 3). There was no significant difference in the zeta potential of the targeted and non-targeted IONP-Tfab constructs, with each showing a near neutral value. HER2 binding of the 30 nm and 100 nm IONP-Tfab conjugates was evaluated using both recombinant protein and live cancer cells. ELISAs with rHER2 protein yielded similar sub-nanomolar EC_50_ values for both 30 nm and 100 nm IONP-Tfab, whereas no binding was observed with the non-targeted 30 nm or 100 nm maleimide-IONPs (IONP-Mal) (Fig. 2A and B). These results demonstrated that Tfab binding affinity was not compromised during IONP conjugation, and that non-targeted IONP-Mal controls had no inherent affinity for the rHER2 receptor. In a similar fashion, both sizes of IONP-Tfab efficiently bound SKBR3 and BT-474 breast cancer cells after 8 hours incubation (Fig. 2C, D). The more efficient targeting of BT-474 is consistent with the marginally higher HER2 expression level of these cells (S4 Fig.). Importantly, Tfab-targeted particles showed no association with MCF7 breast cancer cells (S5 Fig.), which exhibit markedly lower levels of HER2 expression (S4 Fig.). Likewise, neither 30 nm nor 100 nm non-targeted IONP-Mal bound to HER2 positive cells (Fig. 2C, D). Thus, *in vitro* cellular homing of both smaller and larger diameter IONP was critically dependent on the Tfab targeting moiety as well as high levels of HER2 expression.

**Table 3:** Biophysical and biochemical characterization of IONP constructs.

Particle Design	Hydrodynamic diameter (nm)^a^	PDI^b^	Zeta Potential (mV)	Mean Fab/IONP	Mean PEG/IONP
30 nm	24.6	0.152	-0.637	N/A	N/A
IONP-Mal					
30 nm	26.7	0.128	-1.82	N/A	25
IONP-PEG-Mal					
30 nm	30.5	0.148	0.262	10	N/A
IONP-Tfab					
30 nm	35.56	0.184	-0.318	10	25
IONP-PEG-Tfab					
100 nm	86.88	0.156	-0.978	N/A	N/A
IONP-Mal					
100 nm	92.6	0.185	0.252	N/A	350
IONP-PEG-Mal					
100 nm	95.48	0.177	-1.032	90	N/A
IONP-Tfab					
100 nm	102.64	0.224	-0.637	99	350
IONP-PEG-Tfab					

^a^ For convenience, the results and discussion sections refer to only two broad categories of particle sizes: larger “100 nm” particles and smaller “30 nm” particles. The actual diameters of the particles are as shown in this table. Hydrodynamic diameters were measured by dynamic light scattering.

^b^ polydispersity index

**Fig 3:**
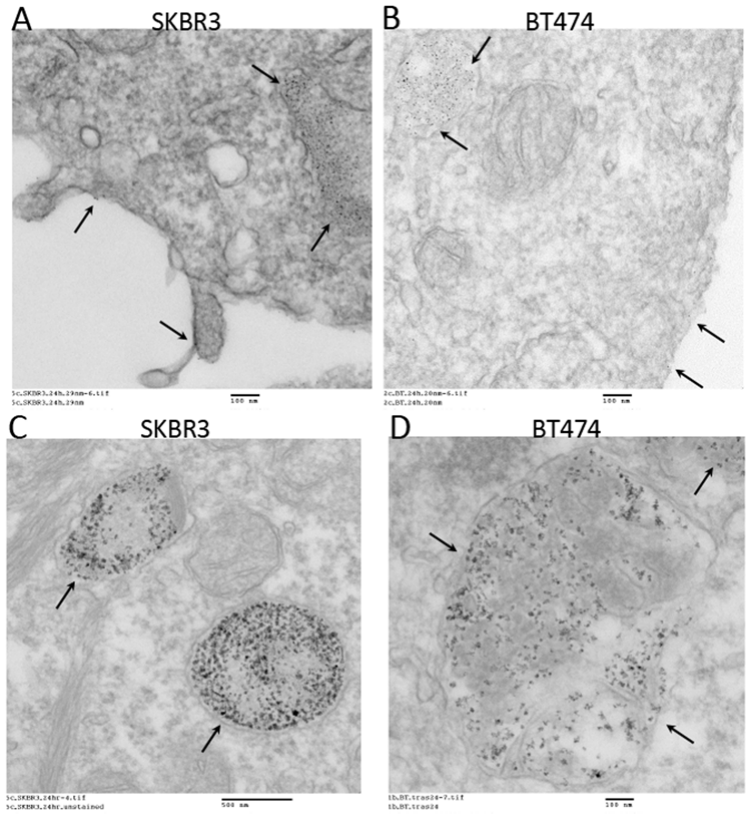
TEM imaging of subcellular localization following *in vitro* binding of IONP-Tfab to HER2+ breast cancer cells. (**A**) At 20,000X magnification in SKBR3 cells, 30 nm IONP-Tfab localize primary to intracellular vesicles with a smaller proportion remaining bound to the cell surface (arrows). (**B**) At 20,000X magnification in BT-474 cells, 30 nm IONP-Tfab exhibit similar localization. (**C**) At 10,000X magnification in SKBR3 cells, 100 nm IONP-Tfab are mainly found in intracellular vesicles (arrows). (**D**) At 20,000X magnification in BT-474 cells, 100 nm IONP-Tfab exhibit similar localization. Scale bars are 100 nm (A, B, D) and 500 nm (C).

To assess the subcellular localization of 30 nm and 100 nm IONP-Tfab conjugates, replicate samples from the cellular ELISA studies were harvested and analyzed by transmission electron microscopy (TEM). The TEM micrographs showed that both 30 nm and 100 nm IONP-Tfab are internalized by BT-474 and SKBR3 cell lines. During 8 hour *in vitro* cellular incubations in complete media, both particle sizes were found to accumulate predominantly within intracellular vesicles, and only a small fraction remained localized on the outside of the cell membrane (Fig. 3).

### *In vivo* tumor targeting of IONP

**Fig 4:**
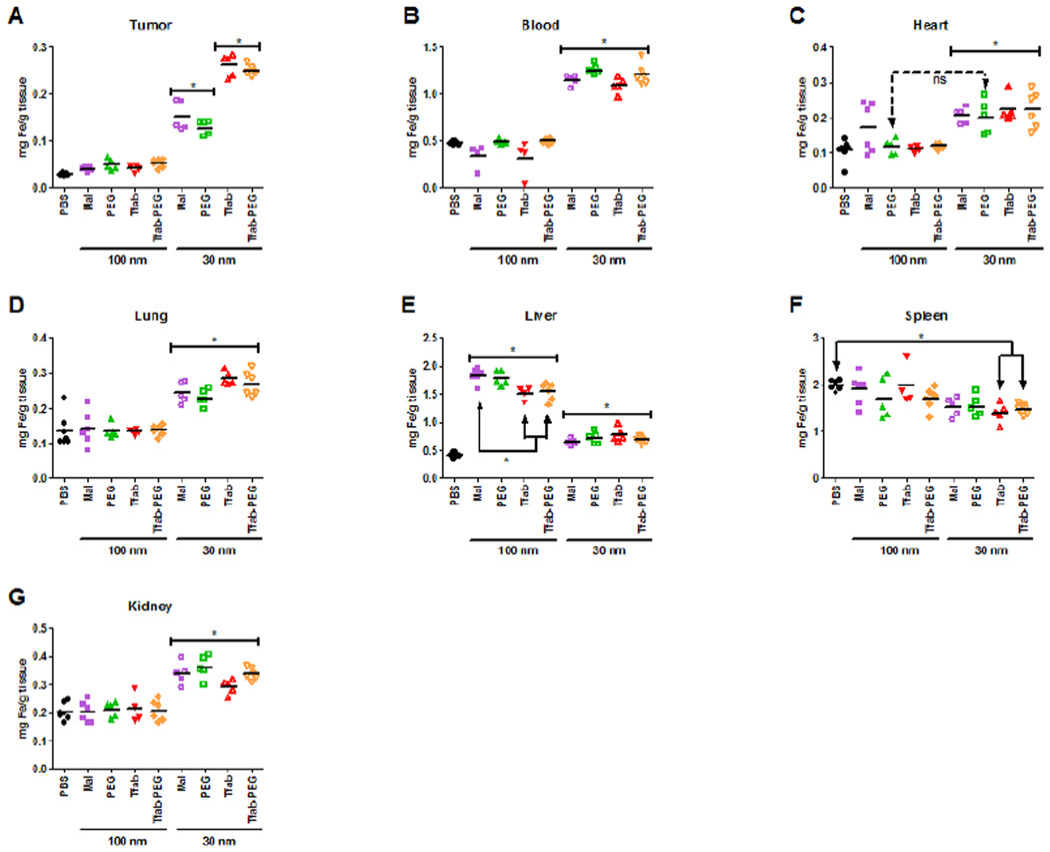
*In vivo* biodistribution of IONP. Nanoparticles (80 μg/g body mass) were systemically administered by tail vein injection in NSG mice bearing BT-474 xenograft tumors. Iron content of various tissue compartments was quantified 24 hours post injection by ICP-MS. (**A**) Tumor, (**B**) Blood, (**C**) Heart, (**D**) Lung, (**E**) Liver, (**F**) Spleen, (**G**) Kidney. Statistical significance (P<0.05) was analyzed by one way ANOVA with a Tukey multiple comparison posttest. Solid brackets with asterisk indicate groups of mice that were not significantly different from each other but were significantly different from other groups outside the bracket. Significant differences between individual groups, including exceptions to the solid brackets, are indicated by solid arrows marked with an asterisk. For the heart, non-significance between individual groups (i.e. exceptions to the solid bracket) is indicated by hatched arrows marked with “ns”. For the heart, the 100 nm maleimide IONP group was not significantly different from any other group in the panel. N = 4 to 7 per group. Statistical analyses were completed using GraphPad Prism 5 (GraphPad Software, Inc., San Diego, CA).

**Fig 5:**
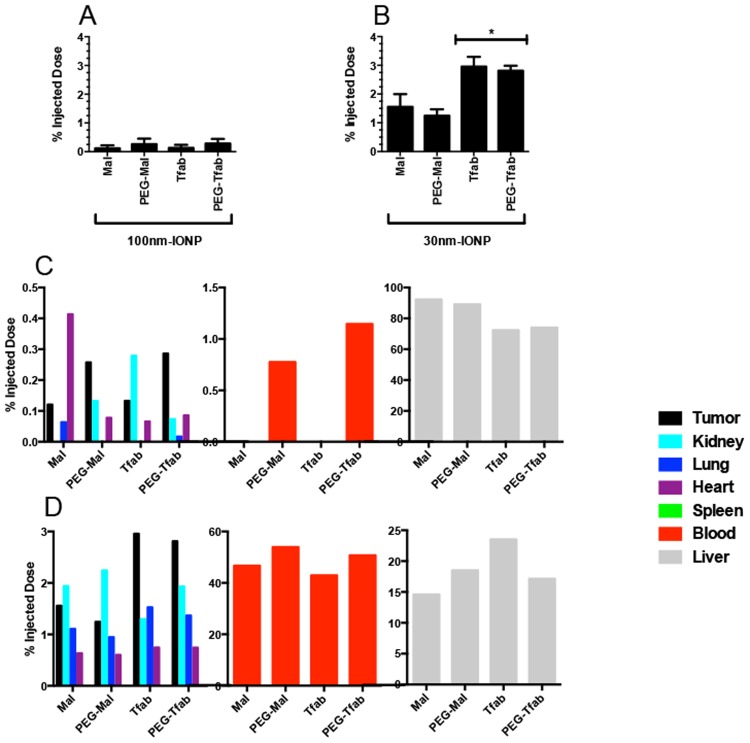
Percent injected IONP dose recovered during i*n vivo* biodistribution studies. Percentages based on raw values from Fig. 4. (**A**) Tumor recovery of 100 nm IONP. Values are not statistically significant relative to background. (**B**) Tumor recovery of 30 nm IONP. (**C**) Recovery of 100 nm IONP from seven different harvested tissues. Liver = grey; blood = red; tumor = black; kidney = cyan; lung = blue; spleen = green; heart = purple. Note the discontinuous y-axis. (**D**) Recovery of 30 nm IONP from seven different harvested tissues. Mean values from N = 4 to 7 mice per group. Error bars in A and B are standard deviation. Statistical significance (P<0.05) was analyzed by one way ANOVA with a Dunnett comparison posttest.

The enhanced *in vitro* performance of targeted 30 nm and 100 nm IONP-Tfab conjugates suggested that they might also exhibit enhanced tumor localization *in vivo*. To test this hypothesis, a single dose (80 μg Fe/g of body mass) of various IONP constructs was administered I/V via the tail vein into NSG mice bearing human HER2 positive BT-474 tumors. Twenty-four hours post injection, tissues were harvested and IONP content was quantified by inductively coupled plasma mass spectrometry (ICP-MS). Consistent with their lack of homing to tumor cells *in vitro*, the non-targeted 100 nm IONP-Maleimide failed to show statistically significant tumor accumulation *in vivo* (Fig. 4A). Tumor accumulation of the 100 nm IONP-Tfab conjugates was similarly negligible, despite the fact that they had exhibited highly efficient HER2 positive cellular targeting *in vitro* and despite the presence of Her 2 overexpression in removed tumors from mice (S8 Fig.). Examination of iron concentration in the blood indicated that both 100 nm IONP-Mal and IONP-Tfab were eliminated from circulation by the 24 hour time point (compare 100 nm groups to PBS, Fig. 4B). For both targeted and non-targeted 100 nm IONP, the only tissue exhibiting significant iron accumulation was the liver (Fig. 4E), where 70–90% of the injected dose was ultimately sequestered (Fig. 5C). Thus, for the 100 nm IONP, the *in vitro* performance advantage of antibody targeting did not translate to enhanced tumor accumulation *in vivo*.

The above results suggested that clearance from the blood might be partially responsible for the poor *in vivo* performance of 100 nm IONP-Tfab. In an effort to reduce liver sequestration and extend circulation times, a PEGylated variant of the 100 nm targeted particles (100 nm IONP-PEG-Tfab) was constructed by covalent attachment of 2 kDa PEG chains (Table 3). *In vitro* assays demonstrated that PEGylation did not alter the rHER2 binding properties of either the targeted or non-targeted IONP (S6B Fig.). Ultimately, however, PEGylation failed to extend blood circulation times, reduce liver sequestration, or exert any other notable effect on *in vivo* biodistribution of 100 nm IONP (Figs. 4, 5A and 5C).

**Fig 6:**
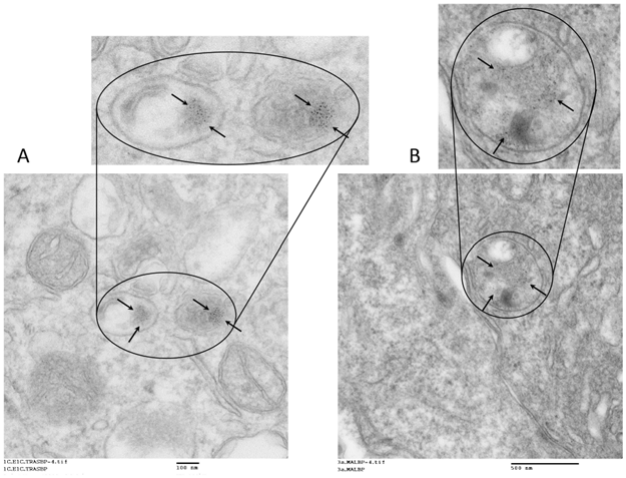
TEM imaging of IONP subcellular localization in mouse tumors. Following systemic administration and homing to BT-474 tumor cells i*n vivo*, 30 nm IONP-Tfab (20,000X magnification) (**A**) and 30 nm IONP-Maleimide (12,000X magnification) (**B**) internalize and concentrate within intracellular vesicles (arrows).

In contrast to the large particles, systemic administration of smaller non-targeted 30 nm IONP-Mal yielded statistically significant concentrations of iron in the tumor (Fig. 4A), averaging 120 μg/g tissue (1.6% of the injected dose, Fig. 5B). Importantly, the addition of the Tfab targeting moiety significantly increased tumor accumulation to 230 μg/g (3% of the injected dose, Fig. 4A and 5B). Thus, in contrast to the larger 100 nm IONP, the *in vitro* performance advantage of antibody targeted 30 nm IONP translated to a 2-fold enhanced tumor accumulation *in vivo*. Compared to the 100 nm IONP, both the targeted and non-targeted 30 nm IONP exhibited significantly elevated blood concentrations at 24 hours (Fig. 4B), and both showed substantially lower concentrations in the liver (Fig. 4E). As seen with the 100 nm IONP, PEGylation of 30 nm IONP did not alter binding to rHER2 *in vitro* (S6A Fig.), but neither did PEGylation influence *in vivo* biodistribution of the smaller IONP constructs (Fig. 4). Finally, it is worth noting that TEM analysis of tumor tissue sections revealed that, as was seen *in vitro*, 30 nm IONPs were internalized by BT-474 cells in the NSG-BT-474 mouse xenograft model (Fig. 6).

Importantly, to minimize variability associated with the experimental procedures, the circulatory systems of mice were not perfused prior to tissue harvesting. As a result, all tissues contained some nominal amount of contaminating blood, and the blood vol% of each tissue contributed proportionally to the measured iron concentrations. In the case of groups receiving various 100 nm IONP, the nanoparticle concentration in the blood was negligible at 24 hours (Fig. 4B), and therefore the blood contribution to tissue iron measurements derived almost exclusively from endogenous hemoglobin. This basal contribution from red blood cells is readily accounted for by the PBS control group. In contrast, all 30 nm IONP exhibited significantly longer circulation times, and in each case more than 40% of the initially injected dose remained in the blood at 24 hours (Fig. 5D). Thus, among mice treated with 30 nm IONP, blood contributed a substantial amount of iron to the total tissue iron concentration.

While the total tumor concentration of IONP is a key factor for many clinical applications, a fundamental objective of this study was assessing the specific tumor tissue association of various IONP constructs. To do so, the biodistribution data was further analyzed by averaging the iron concentration in each tissue of a given treatment group and normalizing that averaged value by the averaged blood iron concentration for the same treatment group (S7 Fig.). While quantification of total iron concentration in the tumor showed that the 100 nm IONP constructs exhibited no statistically significant difference relative to the PBS control group (Fig. 4A), normalizing the tumor iron concentrations of each treatment group with the corresponding blood iron concentrations revealed that there was a general (but not always significant) trend towards low-level tumor tissue accumulation among the various 100 nm IONP groups (S7A Fig.). After normalizing to blood iron concentration, tumor specific accumulation of non-targeted 30 nm IONP-Mal and IONP-PEG-Mal was largely equivalent to that of the 100 nm IONP groups (all ratios ranged between 1.6 and 2.1, S7A Fig.). Thus, the statistically significant total tumor iron concentrations of the non-targeted 30 nm IONP-Mal and IONP-PEG-Mal (Fig. 4A) were derived largely from the blood residing within the tumor as opposed to the tumor tissue itself. In contrast, following normalization to blood iron concentrations, the tumor tissue accumulation of the targeted 30 nm IONP-Tfab and IONP-PEG-Tfab were highly significant relative to the PBS control group (P<0.001, S7A Fig.), and this observation demonstrates that the antibody directed 30 nm IONP specifically localized to tumor tissue following systemic administration in the mouse BT-474 xenograft model.

Considering the impact of blood volume corrections for the other tissue compartments, the various 100 nm IONP constructs showed highly significant and specific liver deposition (S7B Fig.). In contrast, the smaller 30 nm constructs yielded no tissue specific accumulation in the liver (S7B Fig.). Another key insight from this analysis was that none of the eight IONP constructs exhibited tissue specific accumulation with the kidney, spleen, lung, or heart (with one possible exception: heart deposition of 100 nm IONP-Maleimide). Thus, any IONP detected in these healthy tissues appeared to reside within the organs’ fractional blood volume as opposed to specific association with the organ tissue itself.

## Discussion

The magnetic properties of IONP render them highly attractive nanomaterials for imaging and hyperthermia of cancer [36]. However, their clinical utility depends on differential particle accumulation in tumor cells; particles must preferentially partition into tumors versus surrounding healthy tissues. The enhanced permeability and retention effect can yield some tumor accumulation for small particles possessing long circulation half-lives, [37] but such passive targeting may be inadequate for many applications [17,36]. While numerous other IONP studies have examined separately the effects of antibody targeting *in vitro* [29,31,38], antibody targeting *in vivo* [30–32,38], and IONP size *in vivo* [39], the results presented here are the first controlled, systematic, comparative analysis of the *in vitro* versus *in vivo* interplay between IONP size, antibody-mediated molecular targeting, and surface modification with PEG.

The targeted 30 nm and 100 nm IONP-Tfab conjugates were both found to specifically bind to BT-474 and SKBR3 breast cancer cells *in vitro* (Fig. 2). However, with both rHER2 and HER2 postivie cell lines, the total iron accumulation was 2 to 3-fold higher for 100 nm IONP-Tfab versus the smaller 30 nm counterpart. These results are in line with early reports’ showing that cellular uptake of nanoparticles is dependent upon size and shape, both of which affect the membrane-wrapping process [40,41]. Nevertheless, the smaller Tfab antibody fragment employed here yields cellular selectivity similar to that observed with IONP targeted via the full length Tmab IgG [29,42]. In addition to the cellular specificity observed here, the Tfab targeting moiety was found to drive internalization of both 30 nm and 100 nm IONP by HER2 positive breast cancer cells. The intracellular localization of IONP-Tfab is consistent with previous reports that employed the full length Tmab targeting moiety [29,43] as well as more recent studies employing an anti-EGFR single chain antibody fragment[38]. This important phenomenon of cellular internalization could have implications for selective partitioning of IONP into tumors [28] and internalization might also prove critical to therapeutic applications where efficacy is modulated by intracellular localization (*e*.*g*. magnetic hyperthermia and/or cytotoxic drug delivery)[18,44].

While functionalization with Tfab enabled HER2 specific cellular targeting of both IONP sizes *in vitro*, nanoparticle hydrodynamic radius was the key determinant of tumor accumulation *in vivo*. Specifically, the current studies show that 100 nm IONP fail to efficiently access the BT-474 tumor compartment, and active molecular targeting to tumor cell surface antigens was unable to overcome the underlying barriers. Accelerated blood clearance with increasing IONP size is a well-known phenomenon[45], and we speculate that liver sequestration combined with poor extravasation prevented tumor targeting of the larger IONP in the current studies. Interestingly, prior systematic studies with gold nanoparticles showed that larger nanoparticles (60–100 nm) exhibited greater tumor accumulation than smaller nanoparticles (20 nm) [46]. However, a direct comparison with our results is confounded by substantial differences in various experimental design parameters (nanoparticle type, PEGylation, targeting, and perhaps most importantly, tumor cell type and mouse xenograft model).

While the larger IONP failed to show any tumor homing *in vivo*, the smaller 30 nm IONP exhibited statistically significant tumor concentrations, even without the benefit of the Tfab targeting moiety. This is in contrast to prior reports of non-functionalized 30 nm IONP[26], which showed no such specific tumor accumulation. This discrepancy might be explained by differences in the tumor model and/or differences in the properties of the IONP dextran coating, which was a carboxy-terminated PEG in the former study and a mixture of amine and maleimide groups in the current study. As reported here, the *in vivo* tumor homing capacity of smaller IONP was enhanced by the addition of the Tfab targeting moiety, and the extent of 30 nm IONP-Tfab tumor accumulation (13.6% injected dose/g tumor) was equivalent to previous reports of systemically administered 30 nm IONP-antibody conjugates (13.7% injected dose/g tumor) [26,27]. Importantly, in the current study, TEM analysis of excised BT-474 NSG mouse tumors showed that the 30 nm IONP-Tfab retained their cellular-internalizing properties following systemic administration. This fact could have profound implications for IONP-mediated therapy of solid tumors.

In addition to active molecular targeting, IONP surface properties are a known determinant of *in vitro* and *in vivo* performance [47]. For example, particle surface charge has been shown to strongly influence both liver uptake [48] and internalization by cancer cells [49]. However, in the current study, all IONP constructs exhibited highly similar zeta potentials (Table 3), and therefore the observed performance differences among the various IONP were unrelated to surface charge. Functionalization with PEG is another well studied means of manipulating nanoparticle pharmacokinetics [46]. Indeed, PEGylation has recently been shown as an effective strategy for modifying the biodistribution of IONP[50]. In contrast, the *in vitro* and *in vivo* results reported here indicate that PEGylation has little to no effect on IONP performance. This discrepancy with the earlier report is likely attributable to differences in the cancer cell model and the size and number of PEG molecules coupled to the IONP surface. In particular, the prior report estimates PEG loading at several hundred molecules per IONP, whereas comparably sized particles from our study bore a mean of 25 PEG molecules each (Table 3). We did not examine higher loading densities, as our Tfab targeting moieties represented a significant steric barrier to conjugation of additional PEG molecules. Thus, modulating small diameter IONP pharmacokinetics via PEGylation appears to require high surface densities of the conjugated polymer.

## Conclusions

In summary, this systematic study of IONP performance reveals that, *in vitro*, molecular targeting is the key determinant of cancer-specific cellular accumulation, whereas in our BT-474 xenograft mouse model IONP size is the fundamental gatekeeper with respect to *in vivo* tumor targeting. *In vivo*, molecular targeting significantly enhances tumor accumulation for small but not large diameter IONP. Our results suggest that non-targeted 30 nm IONP are efficiently delivered to the tumor by blood flow, but inside the tumor these particles exhibit only low level association with the tumor tissue itself. We hypothesize that the tumor functions as a control volume in dynamic equilibrium, i.e. blood flow continuously carries the non-targeted 30 nm IONP into and out of the tumor at equal rates. Thus, the tumor concentration of these small non-targeted IONP is dictated by their concentration in the blood and the percent blood content of the tumor itself. In contrast, a fraction of the antibody-targeted 30 nm IONP-Tfab is removed from circulation during passage through the tumor and is no longer subject to washout. This fraction of nanoparticles, bound specifically to tumor tissue, explains the enhanced *in vivo* performance of Tfab targeted IONP relative to the non-targeted counterpart. Given the high blood concentrations of 30 nm IONP-Tfab and 30 nm IONP-PEG-Tfab at 24 hours, we speculate that even greater tumor concentrations could be achieved at later time points. We anticipate that these results will prove useful to others seeking to employ systemically administered IONP for treatment or imaging of breast and other solid tumors.

### Supporting information available

Details of cell culture conditions; Tfab purification and purity analysis; antibody competition assays; antibody affinity measurements; cellular binding studies and HER2 receptor profiling; IONP constructs and specificity; *in vivo* biodistribution and immunohistochemistry studies.

## Supporting Information

S1 Fig Characterization of Tfab antibody fragment. ClustalW alignment of Tfab and Tmab light (**A**) and heavy (**B**) chains protein sequences. Cysteines 214 and 223 respectively of Tfab light and heavy chains involved in normal disulfide bonding between light and heavy chain are underlined. Free Cysteine 229 of Tfab heavy chain enabling site-specific conjugation to reactive maleimide groups is also underlined. Identical residues are marked with an asterisks and gaps with a dash. (**C)** Coomassie stained SDS-PAGE gel (C) of purified of Tfab showing in lanes 1–2 reduced Tfab after size exclusion chromatography (SEC) and the same material following conjugation to maleimide-PEG2-Biotin respectively. Lanes 3–4 are, respectively, non-reduced Tfab after SEC and the same material following conjugation to maleimide-PEG2-Biotin. (**D**) Liquid chromatography mass spectrum (LC-MS) of SEC purified of Tfab in reduced form. (**E**) LC-MS of SEC purified Tfab following cysteine activation. (**F**) LC-MS of maleimide-PEG2-Biotin conjugated Tfab. The mass of 23965 corresponds to free light chain conjugated to one maleimide PEG2-biotin molecule (+526 Da). The mass of 25599 corresponds to free heavy chain conjugated to two maleimide-PEG2-biotin molecules (+1052 Da). The mass of 48510 corresponds to intact Tfab conjugated to one maleimide-PEG2-biotin molecule (+526 Da).
(TIF)

S2 Fig Tfab binding affinity and competition against Tmab (Herceptin). (**A**) Representative ELISA binding profiles of Tfab (red) and Tmab (blue) with rHer2 protein. (**B**) Competition ELISA shows Tfab competes with commercial Tmab for binding to human rHer2 protein. (**C**) Tfab binding to both Her2+ (BT474, red; SKBR3, blue) and Her2- (MCF7, dark green; SKOV3, grey; A2780, light green) tumor cells. (**D**) Competition ELISA shows that Tfab competes with commercial Tmab for binding to Her2+ tumor cells (BT474, red; SKBR3, blue). (**E**) Representative ELISA binding profile of Tfab with rHer 2 (red) and rHer3 (Blue) proteins. Error bars represent standard deviation from technical triplicates.
(TIF)

S3 Fig Biolayer interferometry data. Sensorgrams of soluble rHer2 (A-B) and rHer2 and rHer3 (C-D) binding to immobilized Tfab and Tmab (Herceptin) on Fortebio biosensor tips (Streptavidin and recombinant proteinA capture, respectively). Blue curve indicates measured binding kinetics and red line indicates best fit curve from kinetic modeling.
(TIF)

S4 Fig Quantified Her2 expression levels from various cancer cell lines. (**A**) Microsphere beads with different level of FITC fluorescent intensity used to establish calibration curve for flow cytometry. Flow cytometry histograms of (**B**) MCF7, (**C**) BT-474, (**D**) SKBR3, (**E**) A2780 and (**F**) SKOV3 cells after incubation with FITC-maleimide labeled Tfab. Molecules of equivalent soluble fluorochrome (estimated receptor number per cell) were interpolated from the microbead calibration curve.
(TIF)

S5 Fig *In vitro* cellular specificity of IONP constructs. Representative 100 nm IONP-Tfab and 100 nm IONP-Mal binding to Her2 negative (MCF7) and positive (BT-474) tumor cells. Mean values with standard deviation.
(TIF)

S6 Fig *In vitro* rHer2 binding affinity of PEGylated IONP constructs. (**A**) 30 nm IONP-Tfab-PEG (closed circles) and 30 nm maleimide IONP-PEG (open squares). (**B**) 100 nm IONP-Tfab-PEG (closed circles) and 100 nm maleimide IONP-PEG (open squares).
(TIF)

S7 Fig Tissue iron concentration normalized to blood iron concentration for each treatment group. Iron content of various tissue compartments was quantified 24 hours post injection by ICP-MS, and the averaged values from Fig. 4 were normalized to blood iron content from Fig. 4. This normalization corrects for tissue iron content contributed by residual blood within the given tissue compartment. (**A**) Tumor, (**B**) Liver, (**C**) spleen, (**D**) Kidney, (**E**) Lung, (**F**) Heart. Statistical significance (*P<0.05; ** P<0.01; ***P<0.0001) was analyzed by one way ANOVA with a Dunnett multiple comparison posttest to PBS.
(TIF)

S8 Fig Photomicrographs representing immunohistochemistry on tumors excised from murine models. (**A**) Tissue sections of HER2 positive human BT474 breast cancer tumors, and (**B**) MTGB HER2 negative murine adenocarcinoma tumors. HER2 staining in BT474 cells is significantly associated with the cell membrane (brown) but can also clearly be seen in the cytoplasm of many cells. The HER2 negative MTGB cells do not demonstrate any appreciable HER2 staining.
(TIF)

S1 File Supplemental material and methods information. (DOC)
