# Supplementary figures and images for: Tumor Cell Targeting by Iron Oxide Nanoparticles Is Dominated by Different Factors In Vitro versus In Vivo
Source: PLoS One. 2015 Feb 19;10(2):e0115636. doi: 10.1371/journal.pone.0115636 (PMC4335054; doi:10.1371/journal.pone.0115636)

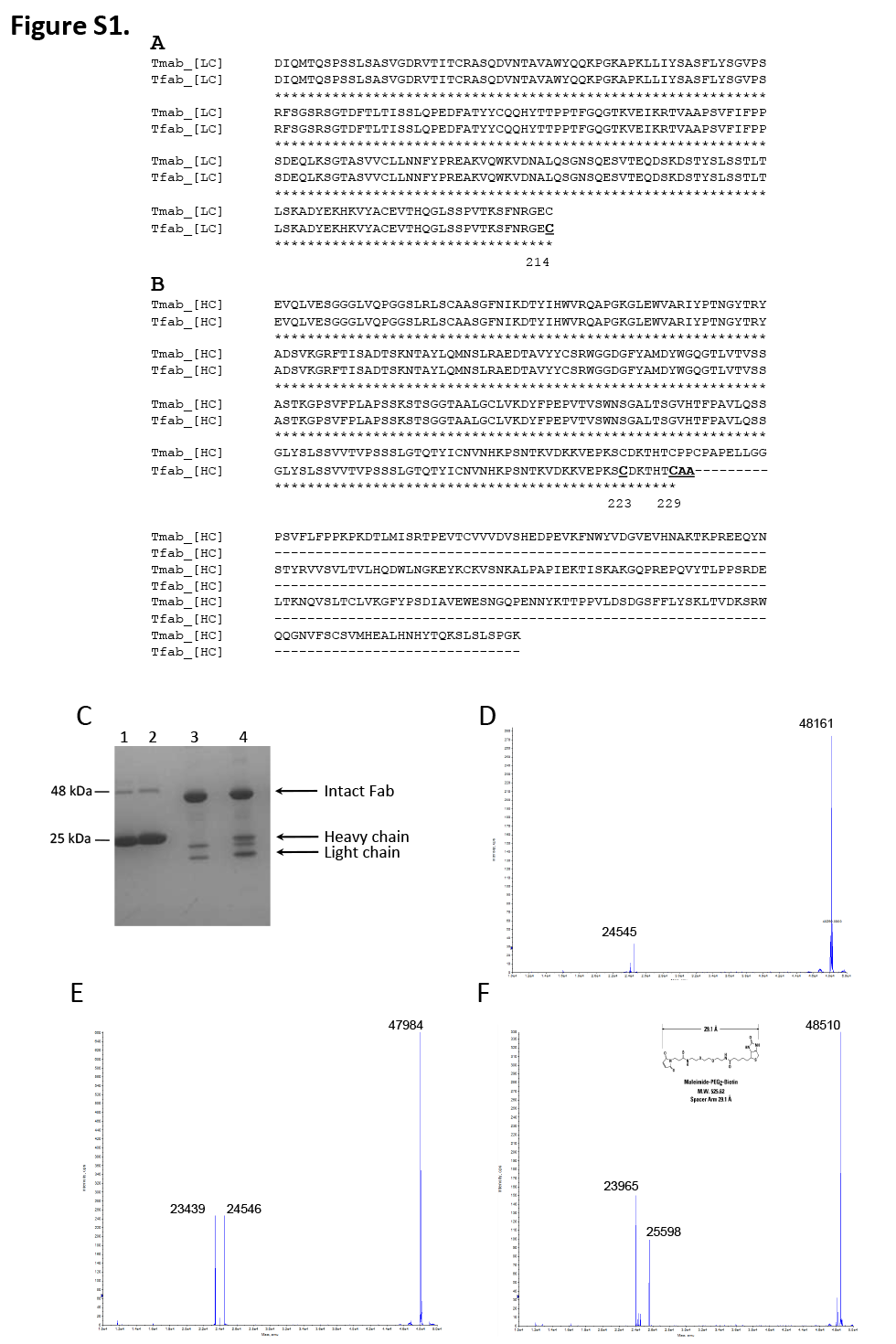

Supplement: S1 Fig — ClustalW alignment of Tfab and Tmab light (A) and heavy (B) chains protein sequences. Cysteines 214 and 223 respectively of Tfab light and heavy chains involved in normal disulfide bonding between light and heavy chain are underlined. Free Cysteine 229 of Tfab heavy chain enabling site-specific conjugation to reactive maleimide groups is also underlined. Identical residues are marked with an asterisks and gaps with a dash. (C) Coomassie stained SDS-PAGE gel (C) of purified of Tfab showing in lanes 1–2 reduced Tfab after size exclusion chromatography (SEC) and the same material following conjugation to maleimide-PEG2-Biotin respectively. Lanes 3–4 are, respectively, non-reduced Tfab after SEC and the same material following conjugation to maleimide-PEG2-Biotin. (D) Liquid chromatography mass spectrum (LC-MS) of SEC purified of Tfab in reduced form. (E) LC-MS of SEC purified Tfab following cysteine activation. (F) LC-MS of maleimide-PEG2-Biotin conjugated Tfab. The mass of 23965 corresponds to free light chain conjugated to one maleimide PEG2-biotin molecule (+526 Da). The mass of 25599 corresponds to free heavy chain conjugated to two maleimide-PEG2-biotin molecules (+1052 Da). The mass of 48510 corresponds to intact Tfab conjugated to one maleimide-PEG2-biotin molecule (+526 Da). (TIF) [file pone.0115636.s001.tif]

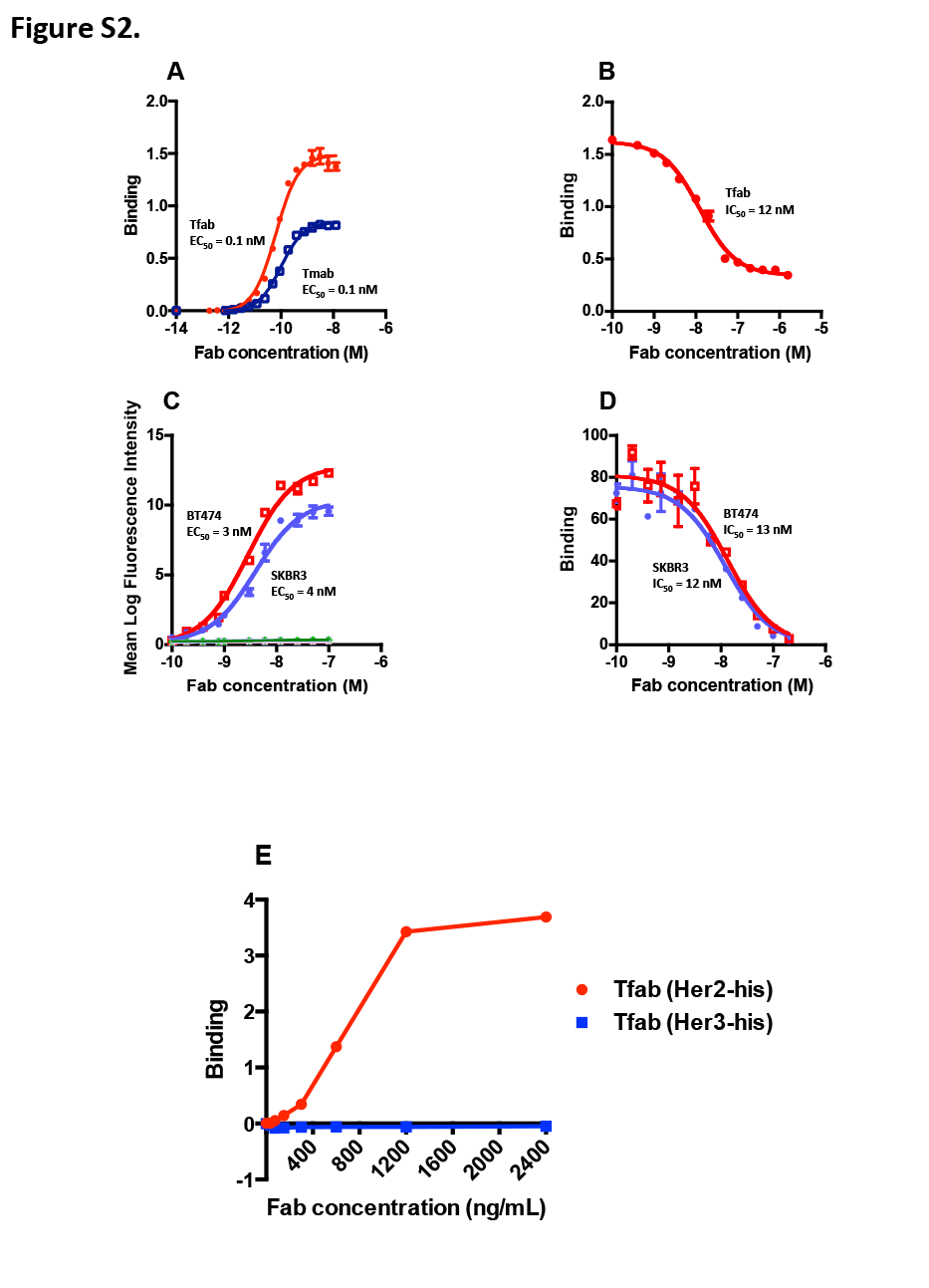

Supplement: S2 Fig — (A) Representative ELISA binding profiles of Tfab (red) and Tmab (blue) with rHer2 protein. (B) Competition ELISA shows Tfab competes with commercial Tmab for binding to human rHer2 protein. (C) Tfab binding to both Her2+ (BT474, red; SKBR3, blue) and Her2- (MCF7, dark green; SKOV3, grey; A2780, light green) tumor cells. (D) Competition ELISA shows that Tfab competes with commercial Tmab for binding to Her2+ tumor cells (BT474, red; SKBR3, blue). (E) Representative ELISA binding profile of Tfab with rHer 2 (red) and rHer3 (Blue) proteins. Error bars represent standard deviation from technical triplicates. (TIF) [file pone.0115636.s002.tif]

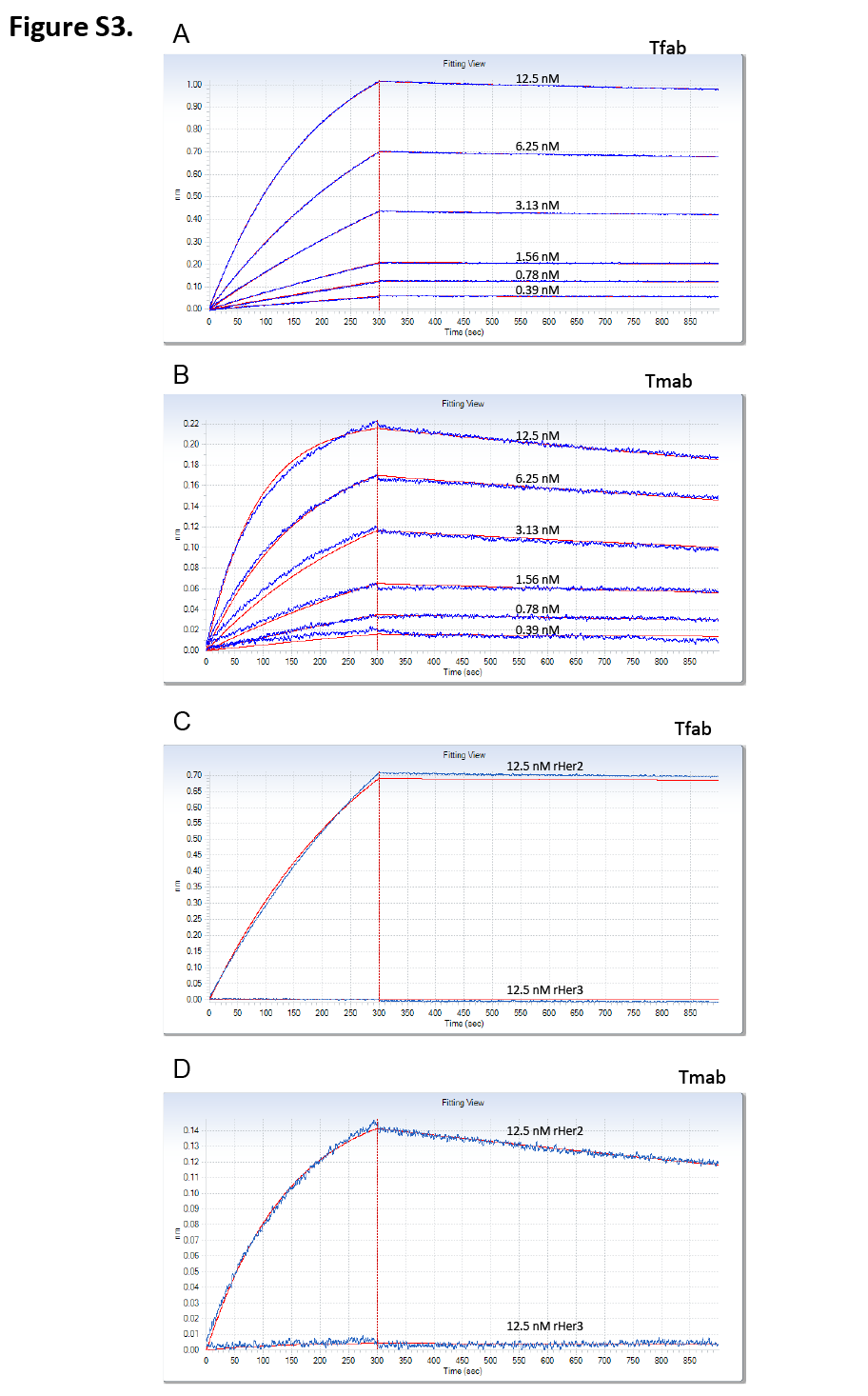

Supplement: S3 Fig — Sensorgrams of soluble rHer2 (A-B) and rHer2 and rHer3 (C-D) binding to immobilized Tfab and Tmab (Herceptin) on Fortebio biosensor tips (Streptavidin and recombinant proteinA capture, respectively). Blue curve indicates measured binding kinetics and red line indicates best fit curve from kinetic modeling. (TIF) [file pone.0115636.s003.tif]

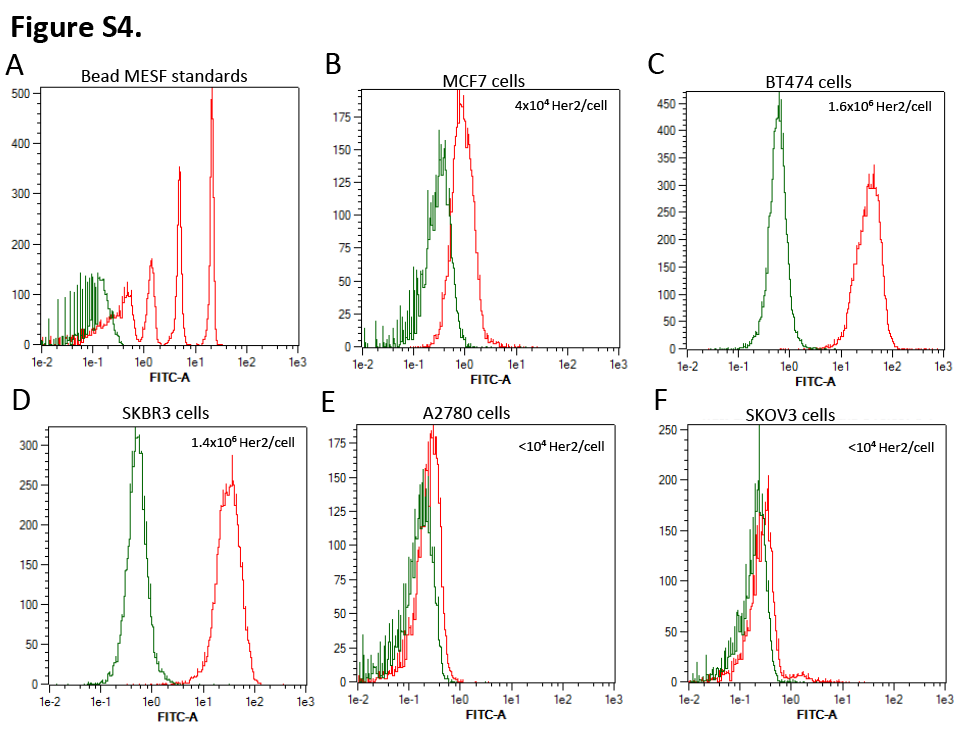

Supplement: S4 Fig — (A) Microsphere beads with different level of FITC fluorescent intensity used to establish calibration curve for flow cytometry. Flow cytometry histograms of (B) MCF7, (C) BT-474, (D) SKBR3, (E) A2780 and (F) SKOV3 cells after incubation with FITC-maleimide labeled Tfab. Molecules of equivalent soluble fluorochrome (estimated receptor number per cell) were interpolated from the microbead calibration curve. (TIF) [file pone.0115636.s004.tif]

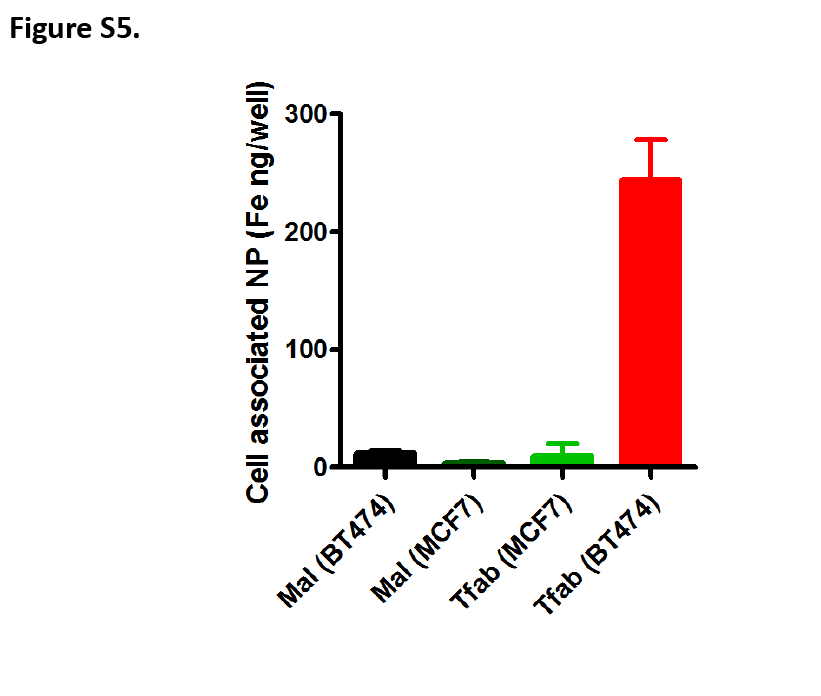

Supplement: S5 Fig — Representative 100 nm IONP-Tfab and 100 nm IONP-Mal binding to Her2 negative (MCF7) and positive (BT-474) tumor cells. Mean values with standard deviation. (TIF) [file pone.0115636.s005.tif]

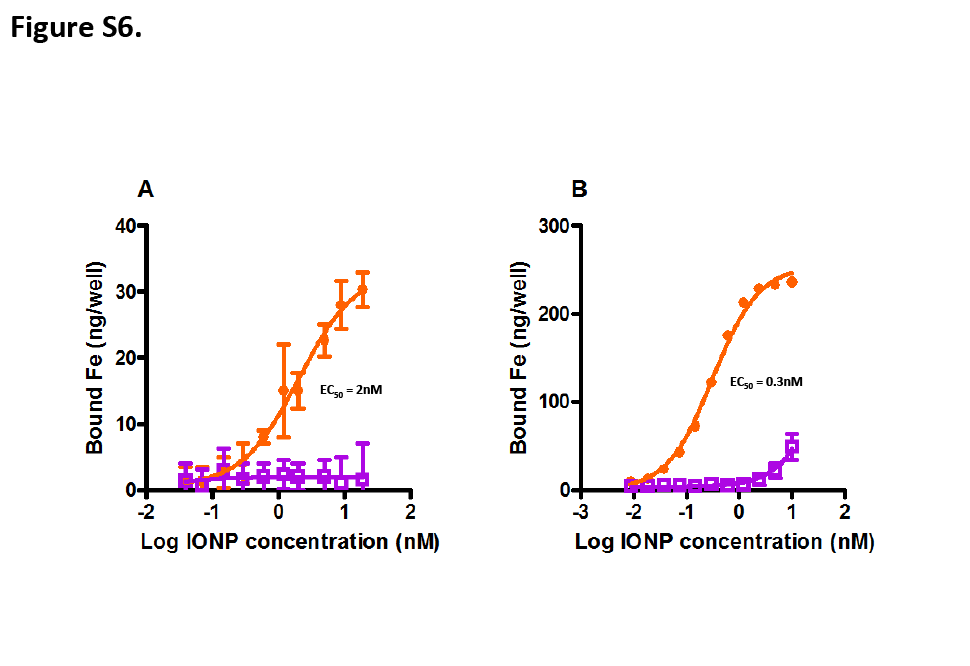

Supplement: S6 Fig — (A) 30 nm IONP-Tfab-PEG (closed circles) and 30 nm maleimide IONP-PEG (open squares). (B) 100 nm IONP-Tfab-PEG (closed circles) and 100 nm maleimide IONP-PEG (open squares). (TIF) [file pone.0115636.s006.tif]

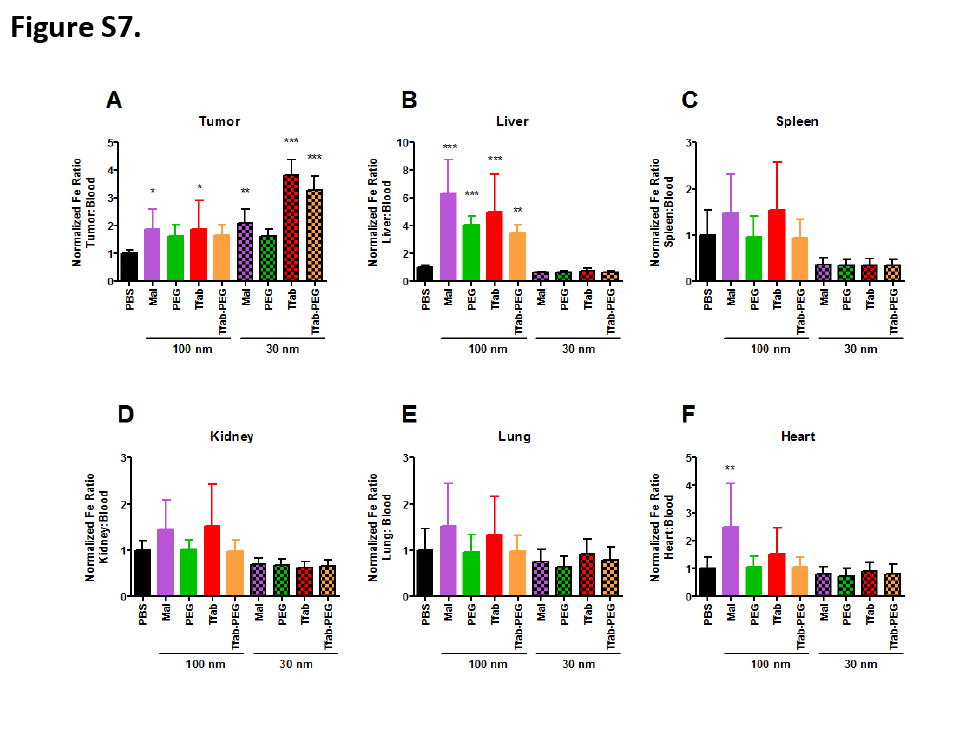

Supplement: S7 Fig — Iron content of various tissue compartments was quantified 24 hours post injection by ICP-MS, and the averaged values from Fig. 4 were normalized to blood iron content from Fig. 4. This normalization corrects for tissue iron content contributed by residual blood within the given tissue compartment. (A) Tumor, (B) Liver, (C) spleen, (D) Kidney, (E) Lung, (F) Heart. Statistical significance (*P<0.05; ** P<0.01; ***P<0.0001) was analyzed by one way ANOVA with a Dunnett multiple comparison posttest to PBS. (TIF) [file pone.0115636.s007.tif]

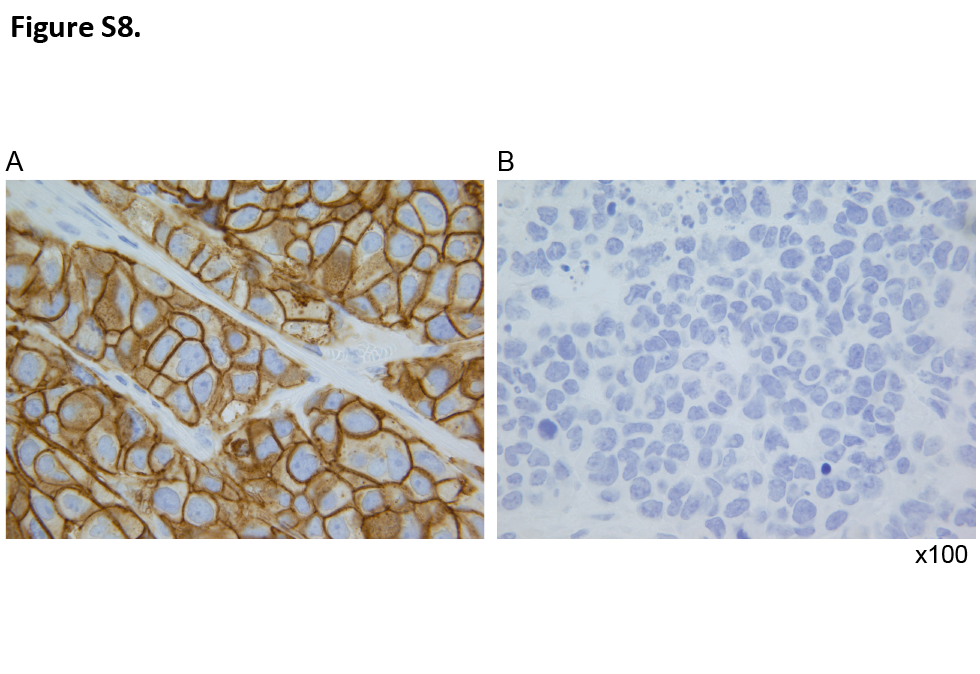

Supplement: S8 Fig — (A) Tissue sections of HER2 positive human BT474 breast cancer tumors, and (B) MTGB HER2 negative murine adenocarcinoma tumors. HER2 staining in BT474 cells is significantly associated with the cell membrane (brown) but can also clearly be seen in the cytoplasm of many cells. The HER2 negative MTGB cells do not demonstrate any appreciable HER2 staining. (TIF) [file pone.0115636.s008.tif]
