# Supplementary material for: Tumor Cell Targeting by Iron Oxide Nanoparticles Is Dominated by Different Factors In Vitro versus In Vivo
Source: PLoS One. 2015 Feb 19;10(2):e0115636. doi: 10.1371/journal.pone.0115636 (PMC4335054; doi:10.1371/journal.pone.0115636)
Supplement: S1 File — (DOC) [file pone.0115636.s009.doc]

**File S1**

**Tumor cell targeting by iron oxide nanoparticles is dominated by different factors *in vitro* versus *in vivo.***

Christian NDong^1^ PhD, Jennifer A.Tate^1^ PhD, Warren C. Kett^1^ PhD, Jaya Batra^1^, Eugene Demidenko^2^ PhD, Lionel D. Lewis^2^ MB BCh.,MD, P. Jack Hoopes^2^ PhD DVM, Tillman U. Gerngross PhD^1,4,5^, Karl E. Griswold PhD^1,3,4*^

^1^ Thayer School of Engineering, Dartmouth, Hanover, NH, USA

^2^ Geisel School of Medicine, Dartmouth, Lebanon, NH, USA

^3^ Program in Molecular and Cellular Biology, Dartmouth, Hanover, NH, USA

^4^ Department of Biological Sciences, Dartmouth, Hanover, NH, USA

^5^ Department of Chemistry, Dartmouth, Hanover, NH, USA

* Corresponding author: Thayer Engineering School, Dartmouth, Hanover, New Hampshire, USA

E-mail address: [karl.e.griswold@dartmouth.edu](mailto:karl.e.griswold@dartmouth.edu)

Phone: 603 646 2127

Fax: 603 646 2277

**Materials and Methods**

**Cells lines, culture conditions**

The human breast cancer cell lines BT-474, SKBR3 and MCF7 and human ovarian cancer cell lines A2780 and SKOV3 were obtained from American Type culture collection (Rockville, MD) and maintained in recommended culture media as suggested by manufacturer. Cells were supplemented with 10% fetal bovine serum (FBS) and penicillin-streptomycin antibiotic at 37^o^C in a humidified atmosphere consisting of 5% CO2 and 95% air. Cell lines were seeded into T150 flasks and grown until confluent. Cells were harvested with 0.25% trypsin, resuspended, and spun down at 1,200 RPM prior to re-suspension and use in subsequent experiments. The FreeStyle 293-F cells were obtained from Life Technologies (Gran Island, NY) and grown in Free Style 293 expression medium (Life Technologies, Gran Island, NY) in a humidified chamber at 37^o^C, 8% CO_2_ in air.

**Expression, extraction and purification of Tfab protein**

Trastuzufab (Tfab) protein sequence was reformatted from its corresponding and commercial full IgG molecule, Trastuzumab (trade name, Herceptin) (Tmab) protein sequence available from literature. Variable and constant regions of the light and heavy chains from Trastuzumab fragment of antibody (Fab) molecule to which we add a free cysteine at C-terminal of the heavy chain were reverse translated, codon optimized for expression in mammalian cells and synthesized by DNA 2.0 Inc. (Menlo Park, CA). CMVR VRC01 expression vectors (NIH AIDS reagent program, Germantown, MD) separately harboring Tfab light chain and heavy chain were co-transfected into suspension HEK 293 cells using polyethylenimine (PEI) (Polysciences, Warrington, PA). Briefly, 250 µg of light chain DNA and 250 µg of heavy chain DNA were combined with 1 ml of PEI and incubated at room temperature for 10 minutes. The mixture was then added to HEK cells in suspension and incubated in a humidified chamber at 37^o^C, 8% CO_2_ for at least 5 -6 days. Secreted Tfab was clarified through centrifugation at 8000 rpm at 4^o^C for 15 min on a Beckman Avanti-J25 centrifuge (Brea, CA). The resulting supernatant was filtered through a 0.45 µm filter to remove any residual cells debris and other large particles before loading onto a FPLC column.

Affinity purification was performed on a pre-packed 5 ml Kappa select column from GE Healthcare (Pittsburgh, PA) as suggested by manufacturer instructions. Final sample was eluted with 100 mM Glycine pH 2.7 in 2 ml eppendorf tubes prefilled with 50 µl of 1M Tris, 5mM EDTA. The purification process was automated on an AKTA FPLC system (GE Healthcare, Pittsburgh, PA). Purified protein was subjected to a second step size exclusion chromatography column using superdex 75 (GE Healthcare, Pittsburgh, PA). Final product was eluted in phosphate buffer saline and stored at -20^o^C until further used.

Purified protein was analyzed using non-reduced and reduced SDS-PAGE conditions and stained with coomassie blue.

**Reductive activation and chemical conjugation of Tfab monoclonal fragment of antibody**

Reduction of purified Fab’ protein was performed by incubation with 20 mM Cysteine at 37^o^C for 15 min. The residual cysteine was removed by buffer exchange hitrap desalting column (GE Healthcare, Pittsburgh, PA) connected to an AKTA FPLC using 50 mM sodium phosphate, 5 mM EDTA pH 6.5. To perform site conjugation, Maleimide-PEG2-Biotin or Fluorescein-5-Maleimide (Thermo Scientific, Rockford, IL) were added to a final concentration of 2mM (10 to 1 molar excess for Maleimide-PEG2-Biotin and for Fluorescein-5-Maleimide to Tfab protein). The conjugation reaction proceeds for 2 hour at room temperature. The excess of Maleimide-PEG2-Biotin and Fluorescein-5-Maleimide were removed by buffer exchange through a hitrap desalting column using PBS pH 7.0 and the resultant Tfabs conjugates (Tfab-Maleimide-PEG2-biotin and Tfab-Fluorescein-5-Maleimide) were stored at -20^o^C and under protected light (for Tfab-fluorescein-5-Maleimide) until further used.

**Purity analysis of Tfab**

Analysis of the monomeric Tfab using SDS-PAGE under reducing conditions (in presence of 50 mM DTT) showed a strong band of ~25 kDa corresponding to Tfab heavy and light chains (Figure S1C in SI). Under non-reducing conditions (absence of DTT), the monomeric Tfab showed the expected 48 kDa band (Figure S1C in SI), but there also appeared two distinct bands with molecular weights corresponding to Tfab heavy and light chains (Figure S1C in SI). This result revealed that the final purified monomeric Tfab fraction is composed of two entities: a majority (~90%) of properly folded Tfab (~48 kDa) and a small (~10%) fraction of Tfab which lack a covalent disulfide bond between cysteine 214 of the light (~23 kDa) and cysteine 223 of the heavy (~24 kDa) chains (Figure S1A in SI).

To confirm these SDS-PAGE results, purified monomeric Tfab was analyzed by liquid chromatography-mass spectrometry (LC-MS) under non-reducing conditions (no DTT). The theoretical mass of properly folded Tfab is 47984 Da, but the observed mass of 48161 Da suggested the presence of an unidentified thiol adduct (most likely from the protein production medium) on the free cysteine (cysteine 229) of the heavy chain (Figure S1D in SI). Following reductive activation with 20 mM cysteine to eliminate this unidentified thiol adduct, the purified monomeric Tfab showed a base peak of 47985 Da, consistent with the expected Tfab molecular mass of 47984 Da (Figure S1E in SI). However, as observed with the non-reducing SDS-PAGE results, the mass spectrum data also revealed the presence of two lower molecular weight entities corresponding to free light (expected = 23439 Da; observed = 23439 Da) and heavy (expected = 24546 Da; observed = 24546 Da) chains of monomeric Tfab (Figure S1E in SI). This result confirmed the presence of both fully form Tfab and a Tfab lacking an interchain disulfide bond (Figure S1A in SI).

In separate experiments, following the 20 mM cysteine activation of monomeric Tfab, the free cysteine (cysteine 229) of the Tfab heavy chain was capped with a hetero bifunctional maleimide-PEG_2_-biotin of 526 Da. Upon reaction with the biotin moiety, the Tfab base peak shifted from 47984 Da to 48510 Da, consistent with the addition of a single maleimide-PEG_2_-biotin molecule (Figure S1F in SI). Additionally, the small fraction of Tfab that lacked an interchain disulfide bond reacted with the maleimide-PEG_2_-biotin as expected. The free light chain peak shifted from 23439 to 23965 Da, corresponding to addition of a single maleimide-PEG_2_-biotin, while the free heavy chain peak shifted from 24546 to 25598 Da, corresponding to the addition of two maleimide-PEG_2_-biotin molecules (one on cysteine 229 and a second on cysteine 223) (Figure S1F in SI). Together, these results demonstrate that the engineered C-terminal free cysteine 229 allows single site specific conjugation to ~ 90% of the monomeric Tfab, while the remaining fraction (~10%) lacking an interchain disulfide bond is subject to reaction with up to two additional maleimide-PEG_2_-biotin molecules.

**Affinity measurement of Tfab and Tmab (Herceptin)**

Tmab (Herceptin) was obtained from Roche (San Francisco, CA). The affinities of Tfab and Tmab protein were analyzed using a biolayer interferometry approach on ForteBio Octet Red instrument (ForteBio, Menlo Park, CA). To measure and compare the monovalent interaction of Tfab and Tmab, commercial recombinants human growth factors receptors 2 and 3 his tagged (rHer2-his and rHer3-his) (AcroBiosystems, Bethesda, MD) were reconstituted at 100 µg/ml (1.4μM) in 1X phosphate buffer saline solution (1XPBS) and loaded in a 96 well plate at different concentration (0.39 – 12.5 nM). Tfab and Tmab were respectively coupled to streptavidin and rproteinA biosensors tips (ForteBio, Menlo Park, CA) at 20 µg/ml and immerse into rHer2-his or rHer3-his. Binding proceeded for 300 seconds before moving the biosensors tips into PBS to monitor dissociation rates. Affinities were analyzed using software provided on the instrument.

**Binding studies and receptor profiling**

The rHer2-his or rHer3-his (AcroBiosystems, Bethesda, MD) was diluted in ELISA coating buffer (100 mM sodium carbonate pH 9.4) to 100 ng/ml (1.4 nM). Two 96-well immulon 4HBX high protein binding plates (Thermo Scientific, Rockford, IL) was coated with 100 µl of rher2-his or rHer3-his at 4^o^C for 16 hours. Coated buffer was removed by aspiration and replace with 300 µl of blocking buffer (0.3% Bovine serum albumin (BSA) (w/v), 0.5% Tween20 (v/v) in 1X PBS pH7.4. Blocking proceed at room temperature for 2hours. Serial dilution (0-100 nM) of Tfab-Maleimide-PEG2-biotin and Tmab in PBS were added and incubate room temperature for 2 hours. Plates were washed 3 times using elisa washing buffer (0.05% BSA (w/v), 0.5% Tween20 (v/v) in 1X PBS. 100 µl of 1/5000 diluted anti streptavidin peroxidase (for Tfab-Maleimide-PEG2-biotin) or 1/100 diluted goat anti-human IgG (Fc) peroxidase conjugated (Thermo scientific, Rockford, IL) in blocking buffer was added to plates and incubated at room temperature for 1 hour. Plates were washed 3 times through ELISA washing buffer and 100 µl of 1-step ABTS (Thermo scientific, IL) was added. The development reaction proceeds at room temperature for 10 minutes and read on plate reader (Spectramax 190, Molecular Devices, Sunnyvale, CA) at 430 nm.

For live cells binding, tumor cells were harvested from T250 flasks (USA Scientific, Ocala, FL) and seeded into a microplate at 50,000 cells per well using PBS containing 2% fetal bovine serum (FBS). Different concentration of Tfab-Fluorescein-5-Maleimide antibody was added to cells and incubated at room temperature on a shaker for 1 hour. Plates were centrifuge at 1200 rpm for 5 min and washed with 2 times with cold PBS containing 2% FBS using a vacuum unit. Cells were resuspended in PBS, 2% FBS and analyzed on a MacsQuant instrument (Miltenyi Biotec, Auburn, CA).

For receptor profiling MCF7, BT-474 and SKBR3 cells were incubated with 100 nM of Tfab-Fluorescein-5-Maleimide for 1 hour in PBS containing 2% fetal bovine serum (FBS). Cells were washed with PBS and analyzed on a MacsQuant instrument (Miltenyi Biotec, Auburn, CA). Molecules of equivalent soluble fluorochrome (MESF) units were calculated using a calibration curve quantum FITC-5 MESF from Bang Laboratories (Fishers, IN) as suggested by the manufacturer.

**Competitive binding studies**

The ability of Tfab-Maleimide-PEG2-biotin to compete with commercial Tmab (Herceptin) for binding the extracellular domain IV of Her2 was investigated with the recombinant protein and live cell based binding. For competitive recombinant protein elisa, 96 well plate was coated with 100 µl of a 100 ng/ml rHer2 (1.4 nM) (Acro Biosystems, Bethesda, MD) in 100mM sodium carbonate pH 9.4 at 4^o^C for overnight. Coated plate was washed 3 times with washing buffer (see above) to remove unbound protein. Plate was blocked with 300 µl of blocking buffer at room temperature for 2 hours. After removing blocking buffer, 50 µl of 400 ng/ml (2 nM) Tmab and 50 µl of trastruzufab-Maleimide-PEG2-biotin at different concentration (0-100 nM) were simultaneous added to the plate for competition. The binding proceeds at room temperature for 2 hours. Plate was washed with 300 µl of washing buffer and 100 µl of 1/3000 diluted goat anti-human IgG (Fc) peroxidase conjugated (Thermo Scientific, Rockford, IL) in blocking buffer was added and the plate incubate at room temperature for 1 hour. Plate was washed 3 times with washing buffer and 100 µl of 1-step ABTS (Thermo Scientific, Rockford, IL) was added to plate and incubate at room temperature for 10 minutes. The signal was read at 430 nm.

For competitive cell binding was perform as described above with cell binding assay with slight modifications. Cells were seed at 50 000 cells per well and 50 µl of 400 ng/ml (2nM) Tmab and different concentration of Tfab-Maleimide-PEG2-biotin antibody (0-200 nM) were added simultaneously to the plate for competition. After 1 hour incubation on a shaker, cells were washed 3 times with cold PBS 2%FBS. 100 ul of 1/200 mouse anti-human IgG (Fc) (Southern Biotech, Birmingham, AL) was added to plate and incubate at room temperature for 1 hour. Cells were washed again 2 times and resuspended in PBS with 2% (v/v) FBS and analyzed on a MacsQuant instrument (Miltenyi Biotec, Auburn, CA).

**Tfab conjugation to 30 nm and 100 nm iron Oxide Nanoparticles (IONPs) and Zeta potential measurements**

Prior to use the 30 nm IONPs were purified by size exclusion chromatography using a Toyopearl HW55F column (600 x 26 mm) eluted at 2ml/min with PBS. 30 nm IONP containing fractions were concentrated by TFF using a 50 kDa hollow-fiber cartridge (Spectrum Labs, Rancho Dominguez, CA). The 100 nm IONPs were purified using Macs separation LS columns (Miltenyi Biotec, Auburn, CA) and eluted with sterile water. To perform site conjugation on 30 nm and 100 nm IONPS, Sulfo GMBS (Thermo Scientific, Rockford, IL) was added to IONPs in 100 fold molar excess for sulfo-GMBS and incubated at room temperature for 2 hours in 100 mM sodium phosphate buffer pH 7.2. The excess of sulfo-GMBS was removed by buffer exchange with 30 mm MES, 5 mM EDTA pH 6.5 using hitrap desalting column and concentrated by TFF. Tfab was reduced with 20 mM cysteine and buffer exchange with 30 mM MES, 5 mM EDTA pH 6.5 to remove the excess of cysteine using hitrap desalting column. Cysteine reduced Tfab was added to the activated IONP at an equal w/w ratio and incubated at room temperature for 16 hours at 4^o^C on a shaker set at 125 rpm. The unbound protein from 30 nm IONP reactions was removed by size exclusion chromatography using Toyopearl HW55F on an automated AKTA FPLC and then concentrated by TFF. The unbound protein from 100 nm IONP reactions was removed using Macs separation LS columns (Miltenyi Biotec, Auburn, CA). For PEGylation, PEG thiol (Laysan Bio, AL) average molecular weight was reduced with TCEP and purified by RPLC HPLC and lyophilized prior to use. The purified material was stored at -20 °C. PEG was assayed by the barium chloride/iodine method.[^35^](#_ENREF_35)[_ENREF_34](#_ENREF_34) Mixed PEGylated Tfab and IONPs were prepared as described for non-PEGylated IONPs, except after the addition of Tfab the mixture was shaken at 4 °C for 2 hrs before 0.5 equivalents w/w of PEG thiol/Tfab was added and the mixture shaken at 4 °C for an additional 16 hrs. An equal amount of PEG thiol was added to a similar aliquot of freshly prepared maleimide-IONPs. The IONPs were purified as described above. All process was performed in a sterile environment using sterile and endotoxin free buffers.

The hydrodynamic Z-average diameters (HDD) and zeta potentials (mV) of small and large IONP-Tfab conjugates were measured using a Zetasizer Nano ZS (Malvern Instruments, UK). For measuring the HDD the IONPs were diluted to 0.05 mg/ml in PBS. For the determination of zeta potential the IONPs were diluted to a concentration of 0.2 mg/ml in 10 mM NaCl.

**Quantification of number of Tfab per IONPs**

Micro BCA assay (Thermo Scientific, Rockford, IL) was used to determine the amount of Tfab covalently bound to 30 nm and 100 nm IONP-Tfab constructs. Similarly, the concentration of non-conjugated Tfab in the reaction supernatant was also determined by BCA. Bovine serum albumin (BSA) standards (from 0-70 µg/ml) and unconjugated IONPs standards (from 0-60µg/ml) were prepared in 150 µl total volume using PBS as diluent. BSA standards, unconjugated IONPs standards, Tfab conjugated 30 nm IONPs sample and unconjugated Tfab were added in a 96-well plate assay in triplicates and immediately read at 480 nm using a plate reader (Spectramax 190, Molecular Devices, Sunnyvale, CA). After reading, 150 µl of prepared micro BCA assay was added to each sample and the plate was incubated at 37^o^C for 2 hours before reading at 562 nm using a plate reader. Tfab-IONP concentration was calculated from the derived standard curve of uncoated IONPs read at 480 nm and used to calculate the corresponding absorbance value using the uncoated IONP standard curve read at 562 nm. The net absorbance of Tfab conjugated to IONP was determined by subtracting the calculated absorbance versus the measured absorbance value at 562 nm. Net absorbance value was converted into a number of BSA equivalents using the BSA standards curve at 562 nm. The concentration of Tfab on IONP was determined by dividing the net BSA equivalent by relative response factor determined for unconjugated Tfab (1.75). Finally, number of Tfab per IONP was calculated by dividing the concentration of Tfab on the surface of IONP by the concentration of IONP in solution (obtained with the uncoated IONP standard curve read at 480 nm).

**30 nm and 100 nm Tfab functionalized Nanoparticles binding studies**

The rHER2-his (AcroBiosystems, Bethesda, MD) and cells (SKBR3 and BT-474) were used for binding studies procedures of 30 nm and 100nm Tfab functionalized nanoparticles. A 96-well immulon 4HBX high protein binding plates (Thermo scientific, Rockford, IL) was coated with 100 µl of rHER2-his (diluted in PBS at 2µg/ml) and incubated at 4^o^C for 16 hours. Coated buffer was removed by aspiration and replace with 300 µl of blocking buffer (2% (BSA) (w/v) in 1X PBS pH7.4. Serial dilutions (0 - 30 nM) of 30 nm IONP-Tfab or 100 nm IONPs-Tfab (0-5 nM) in sample diluents (0.1% BSA (w/v) in PBS pH 7.4) was added and incubates at room temperature for 1 hour. Plates were washed 3 times using washing buffer (Tris buffer saline (TBS), 0.05% Tween20 (v/v)). Nanoparticles uptake was measured using a ferrozine-based iron assay adapted from Reimer *et al.*, 2004. After washing, 100 µl of 1.4 M HCl was added to each well and plate were sealed and heated at 70^o^C for 2 hours. Plate was centrifuged at 200 rpm to settle liquids and 100 ul of ferrozine reagent (6.5 mM ferrozine, 13 mM neocuproine, 2M ascorbic acid diluted in 5M ammonium acetate) were added to each well and shake for 5 minutes. Plates were read at 562 nm on plate reader (Spectramax 190, Molecular Devices, Sunnyvale, CA) and amount of iron per well calculated by using a standard curve of iron (FeCl_3_) identically process as samples.

For cellular binding, SKBR3 and BT-474 were seeded at 100 000 cells per well on a 48-well plate (Corning, Tewksbury, MA) and allowed to attach overnight. Old medium was removed and replaced with 300 µl of fresh medium containing serial dilutions of 30 nm IONP-Tfab or 100 nm IONP-Tfab, and the suspensions were incubated at 37^o^C for 8 hours. Unbound nanoparticles were removed by washing 3 times with PBS. Cells were treated with 100 µl of sodium hydroxide (NaOH) and place on a shaker in 37^o^C room for 1 hour. The ferrozine assay was performed as described above, except 300 µl of each reagent was added.

**Tissue Harvesting and ICP-MS digestion**

24 hours after IONPs injection, mice were euthanized according to approved protocol and checked for pain stimulus response by pinching the leg before collecting tumor tissues. Removed tissues were placed on a pre-weighed weigh boat for massing, using clean instruments between dissections. After weighing, part of the tumor tissues were collected and placed in a vial containing an excess of 10% formalin for histology analyses. Tissues were allowed to fix for at least 24 hours at room temperature before submitting to the Pathology Translation Research Core at Dartmouth. Tissues were subjected to standard block processing, with adjacent sliced-slides stained with hematoxylin and eosin (H&E), and Prussian blue with Nuclear Fast Red. The remaining part of collected tumor tissues were placed in a pre-weighted 15ml conical tube (Sarstedt, Newton, NC) and post-weighed to determine remaining tissues mass. For transmission electron microscopy, tumor tissues were slice into equally sized slices and placed in a petri dish filled with primary fixative solution (3% glutaraldehyde; 1% paraformaldehyde in 0.1M sodium cacodylate, pH 7.4) and incubate at room temperature for 10 minutes. After incubation, tumor slices were further cut down into 2x2x2 mm pieces, transferred to a vial containing fresh fixative solution and fixed for an additional 24 hours at 4^o^C. Fixed tissues were submitted to the Dartmouth Medical Electron Microscope Facility for imaging.

For iron content determination, tumor tissues were digested with a 1:3 volumetric mixture of hydrochloric acid to nitric acid (Fisher Scientific, Pittsburgh, PA) at room temperature for 1 hour. A reference sample was processed and digested in concert with tumor samples. Following digestion, samples were then incubated at 70˚C for two hours with frequent shaking. After heating, samples were allowed to cool at room temperature before submitting to the Dartmouth Trace Elements core for iron content analysis.

**Immunohistochemistry (IHC)**

4μm thick paraffin sections were cut and mounted on silanized slides. Heat-induced antigen retrieval was performed in a water bath, and automated IHC staining with the HER2/ErbB2 rabbit monoclonal antibody (Abcam, Burlingame, CA) diluted at 1:100 was performed on a Biogenix i-6000 automated immuno-stainer in accordance with the manufacturer’s instructions.
